# Exploiting nanopore sequencing advances for tRNA sequencing of human cancer models

**DOI:** 10.1093/narcan/zcaf044

**Published:** 2025-11-03

**Authors:** Adva Kochavi, Arno Velds, Maya Suzuki, Shinichiro Akichika, Tsutomu Suzuki, Roderick L Beijersbergen, Reuven Agami

**Affiliations:** Division of Oncogenomics, Oncode Institute, The Netherlands Cancer Institute, Plesmanlaan 121, 1066CX Amsterdam, the Netherlands; NKI Genomics Core facility, The Netherlands Cancer Institute, Plesmanlaan 121, 1066CX, Amsterdam, The Netherlands; Department of Chemistry and Biotechnology, University of Tokyo, 7-3-1 Hongo, Bunkyo-ku, Tokyo 113-8656, Japan; Department of Chemistry and Biotechnology, University of Tokyo, 7-3-1 Hongo, Bunkyo-ku, Tokyo 113-8656, Japan; Department of Chemistry and Biotechnology, University of Tokyo, 7-3-1 Hongo, Bunkyo-ku, Tokyo 113-8656, Japan; NKI Genomics Core facility, The Netherlands Cancer Institute, Plesmanlaan 121, 1066CX, Amsterdam, The Netherlands; Division of Molecular Carcinogenesis, The NKI Robotics and Screening Center, The Netherlands Cancer Institute, Plesmanlaan 121, 1066CX Amsterdam, The Netherlands; Division of Oncogenomics, Oncode Institute, The Netherlands Cancer Institute, Plesmanlaan 121, 1066CX Amsterdam, the Netherlands; Erasmus MC, Rotterdam University, Wytemaweg 80, 3015 CN, Rotterdam, the Netherlands

## Abstract

Transfer RNAs (tRNAs) are essential regulators of protein synthesis, and dysregulation of their abundance and modification status is involved in many human diseases including cancer. Despite the rapid development of novel tRNA sequencing approaches, due to tRNAs’ stable secondary structure and abundant modification sites, the human tRNA landscape has remained mostly unexplored. Here, we evaluated the new RNA004 chemistry of Oxford Nanopore Technologies, that is integrated with updated Dorado base-caller models, for tRNA quantification and modification annotation in human cancer models. We demonstrated that this technology identifies variations in tRNA expression across cancer cell lines and in response to external stress conditions, with highly reproducible results. We also show that analysis of base-calling error rate can indicate the presence of known modifications, including the cancer-associated tRNA^Phe^-Wybutosine modification. Furthermore, implementing the updated Dorado modification-calling feature, we showed the potential of RNA004 tRNA-seq in predicting common tRNA modifications. We also pinpointed possible limitations and challenges associated with both modification calling methods. Overall, RNA004 tRNA-seq can potentially enhance our understanding of the human tRNAome by simultaneously analyzing both tRNA abundance and modifications.

## Graphical Abstract

**Graphical Abstract:**
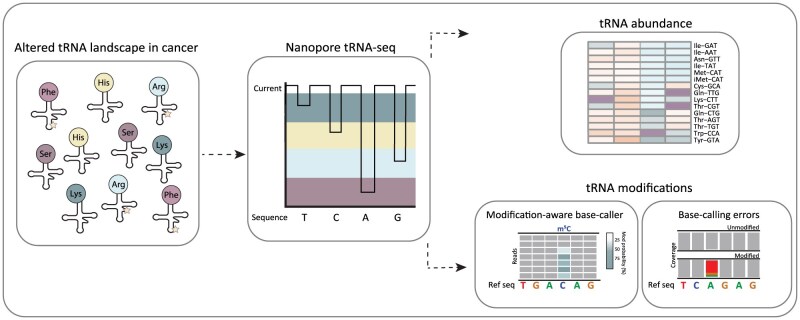

## Introduction

Transfer RNAs (tRNAs) are dynamic adapters, matching the appropriate amino acid to its cognate codon and are critical for protein production. Recent studies have uncovered the importance of tRNA dynamics in redirecting gene expression in response to environmental changes and cellular requirements [1–4]. Not surprisingly, alterations in the abundance of tRNAs and their modifying enzymes were found in many cancers, supporting the translation of transcripts enriched in proliferation and metastasis-regulating genes. tRNA abundance is modulated in cancer via oncogenic signaling, regulation of transcription, or via tRNA-modifying enzymes that are important for tRNA structure, stability, and decoding accuracy [5–7].

tRNA profiles were shown to be significantly different not only between normal tissues and tumor tissues but also between tumor types [7–9]. Alterations of tRNA-modifying enzymes were also found to play a major role in cancer. For instance, epigenetic loss of tRNA wybutosine-synthesizing protein 2 (TYW2) in colon cancer is linked to enhanced epithelial-to-mesenchymal features and poor survival outcomes [10].

The above reports establish tRNAs as predictive biomarkers for cancer and highlight the importance of developing novel technologies for quick and efficient detection of tRNA abundance and modifications in cancer cells. Indeed, many innovative next-generation sequencing (NGS) technologies were developed to efficiently quantify eukaryotic tRNA abundances [11–14]. Despite the success of these strategies in accurately quantifying tRNA pools, these approaches have some major limitations. As the library preparation process depends on reverse transcription (RT) enzymes for conversion of tRNA to complementary DNA (cDNA), followed by polymerase chain reaction (PCR), it creates sequencing biases of highly modified tRNAs, as these modifications interfere with the Watson–Crick base pairing and the RT process. Incomplete PCR can also introduce bias against specific tRNA sequences [15]. And most importantly, many of these methods include the removal of modifications for accurate RT, leading to loss of information and the inability to detect transcript-specific modifications. To address these limitations, newly developed methods utilize the direct RNA sequencing (DRS) platform of Oxford Nanopore Technologies (ONT) to sequence tRNAs [16–19]. Using this platform, native tRNA molecules translocate through nanopores, inducing interruptions in the electric current that are translated to RNA sequence in real-time. Thus, tRNA abundance and modifications can be potentially inferred simultaneously [16–19].

So far, nanopore tRNA-seq methods have been developed and optimized for bacteria [17] and yeast models [16, 18]. tRNA libraries were prepared using nanopore DRS Kit with RNA002 chemistry (SQK-RNA002), and double adapter ligation steps were performed to improve the sequencing yield of mapped tRNAs. Adjustment of nanopore default base-calling settings was also important, as shorter RNA molecules could not be sequenced properly using the default settings of RNA002 chemistry. While these approaches seemed to improve sequencing results, RNA002 chemistry still suffered from low output and mapping quality due to the short and complex structure of tRNAs [20, 21].

As the human tRNA landscape is highly complex compared with previously examined models, consisting of >400 potential tRNA genes with an average of 13 modifications per tRNA [22], improving sequencing yield is necessary for accurate human tRNA sequencing. Recently, ONT released a new direct RNA sequencing chemistry, RNA004, which includes improved RNA-compatible pores, yields higher RNA read count output compared to RNA002, and allows the sequencing of RNA molecules <50 nucleotides. ONT also released a complementary updated base-caller (Dorado) for *de novo* calling of common RNA modifications [21].

In this study, we optimized and applied tRNA sequencing using the new RNA004 chemistry to assess tRNA repertoire in human cancer models. We successfully captured six times more mapped reads while significantly reducing antisense mapping (proxy for mis-mapping) in comparison to the previously established method [16]. Using this method, we collected reproducible tRNA expression profiles for different cancer cell lines and identified tRNA abundance alterations following specific stress exposures. We further evaluated tRNA modifications using two modification detection methods. As modifications often interfere with anticipated current signals [16, 17], we analyzed base-calling errors to deduce established modification sites and evaluate variations in the cancer-related modification wybutosine. Given that not all modifications introduce base miscalls, for a more comprehensive view of tRNA modifications, we implemented the new Dorado modification-calling algorithm. We confirmed high modification probabilities of common tRNA modifications, 5-methylcytosine (m^5^C) and pseudouridine (Ψ), at their conserved positions and previously annotated sites. Despite these findings, we highlighted the challenges associated with these types of modification analysis, emphasizing the necessity for further validations and benchmarking to improve accuracy.

## Materials and methods

### Cells and reagents

PC3 and DU145 cells were cultured in Roswell Park Memorial Institute 1640 Medium (RPMI 1640, Gibco) supplemented with 10% fetal bovine serum (Gibco), 100 U/ml penicillin–streptomycin (Gibco). HEK293T, A375, SK-MEL-28, SNB-19, HT-29, and MDA-MB-231 cells were cultured in Dulbecco’s modified Eagle’s medium (DMEM, Gibco), supplemented with 10% fetal bovine serum and 100 U/ml penicillin–streptomycin. All cell lines were maintained in a humidified atmosphere containing 5% CO_2_ at 37°C and were regularly tested by PCR analysis and were found negative for mycoplasma contamination. Arginine deiminase (ADI) was used at a final concentration of 250 ng/ml and etoposide (Selleckchem) was used at a final concentration of 10 μM. Polyethylenimine (PEI, Polysciences) was dissolved in water at concentration of 1 mg/ml. All treatments were given for 48 h before downstream analyses.

### Cloning

TYW2 sequence was amplified from TYW2-PLX304-blast plasmid of broad library of open reading frames (ORFs) using primers listed in the Supplementary Data Table S1. Then, the PCR product was digested using the introduced restriction sites of XbaI and NotI restriction enzymes, ran on a 1% agarose (Merck) gel and purified using the “Wizard SV Gel and PCR clean-up system” (Promega). The sequence was then ligated in the XbaI and NotI sites in the pCDH-puro vector using T4 DNA ligase (Thermo Scientific) for 1 h at room temperature. All resulting plasmids were sequence verified by Sanger sequencing (Macrogen).

pLentiCRISPRv2-puro plasmid was digested using FastDigest Esp3I and dephosphorylated with FastAP enzymes (both from Thermo Scientific). Then, the digested vector product was run on a 1% agarose (Merck) gel and was purified using the “Wizard SV Gel and PCR clean-up system” (Promega). Oligonucleotides targeting TYW2 or URM1 (Supplementary Data Table S1) were annealed and phosphorylated using T4 PNK. The digested vector and the annealed oligonucleotides were ligated using T4 DNA ligase (Thermo Scientific) for 1 h at room temperature. Finally, the ligation products were transformed to DH5α competent cells (Invitrogen). All resulting plasmids were sequence verified by Sanger sequencing (Macrogen).

### Lentiviral production and transduction

For the production of lentiviruses, HEK293T cells were seeded in tissue culture dishes and transfected the next day. For each transfection, 10 μg of the lentiviral backbone of interest, 5 μg of pMDL RRE, 3.5 μg pVSV-G, and 2.5 μg of pRSV-REV plasmids were mixed. For TYW2 complete deletion 5 μg of each of two lentiviral knockouts plasmids were mixed for total of 10 μg (as previously described [10]). Next, 63 μl of a 1 mg/ml PEI solution was added. After mixing, the solution was left at room temperature for 15 min, after which it was added to the HEK293T cells. The next day, the medium was replaced by fresh medium. The lentivirus-containing supernatants were collected 48 and 72 h after transfection, and snap-frozen in liquid nitrogen. Lentivirus-containing supernatants were supplemented with 8 μg/ml polybrene (Sigma) then used for transduction of the target cells. Twenty-four hours after transduction, transduced cells were selected by the addition of 2 μg/ml puromycin (Adipogen) to the medium.

### Western blotting

Proteins of interest were visualized by sodium dodecyl sulfate–polyacrylamide gel electrophoresis (SDS–PAGE) and western blotting. Cell lysates were prepared by straight lysing in 1× Laemmli buffer. Proteins were separated by SDS–PAGE gels and transferred onto 22-μm pore size nitrocellulose membranes (Santa Cruz). Stainings were performed using TYW2 (Novus Bio, 1:500), Tubulin (Santa Cruz, 1:10 000), and URM1 (ProteinTech 1:1000) antibodies. IRDye 680RD donkey anti-mouse (LI-COR, 926-68072, 1:10 000), IRDye 800CW goat anti-rat (LI-COR, 926-32219, 1:10 000), and IRDye 800CW goat anti-rabbit (LI-COR, 926-32211, 1:10 000) were used as secondary antibodies. Visualization was performed by use of an Odyssey infrared scanning device (LI-COR).

### tRNA extraction and deacylation

For tRNA extraction, total RNA was isolated using Trizol reagent (Invitrogen), according to manufacturer’s instructions. The samples were treated with Turbo DNase (Thermo Fisher Scientific) and small RNA fraction containing tRNAs (≤200 nt) was retained using a Zymo RNA Clean and Concentrator-5 kit (Zymo Research). For deacylation, the retained fraction was incubated in 50 mM Tris–HCl (pH 9.0) at 37°C for 30 min and recovered using a Zymo RNA Clean and Concentrator-5 kit (Zymo Research). The tRNA profiles were confirmed using Agilent 2100 Bioanalyzer, RNA 6000 Nano Assay (Supplementary Fig. S1A).

### tRNA sequencing library preparation

**Figure 1.**
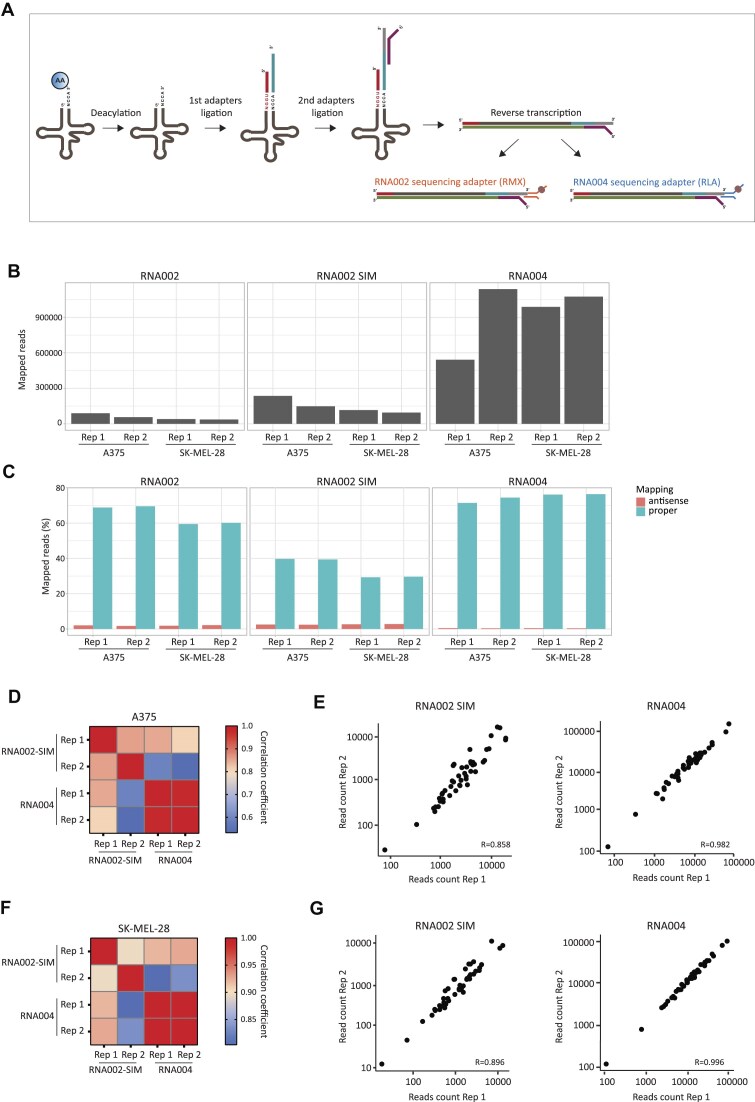
(**A**) Schematic overview of nanopore tRNA-seq using either RNA002 or RNA004 chemistries. (**B** and **C**) Bar plots depicting the number of mapped reads (B) and relative fraction of proper mapped reads (blue) and antisense reads (red) (C) obtained with RNA002 chemistry with default settings, RNA002 chemistry with custom settings (“RNA002 SIM”) or RNA004 chemistry. (**D**–**G**) Heat maps (D and F) and scatter plots (E and G) depicting correlation between two biological replicates of tRNA abundances in A375 (D and E) or SK-MEL-28 (F and G) cell lines sequenced with RNA002 SIM or with RNA004. Each point represents one tRNA isoacceptor type and Pearson’s correlation coefficient is indicated.

tRNAs libraries were prepared as previously described [16] (Fig. 1A) and according to nanopore direct RNA sequencing protocols with some adjustments. The libraries were prepared using either SQK-RNA002 kit (ONT) or SQK-RNA004 kit (ONT) and all oligos used were acquired from Integrated DNA Technologies (IDT). In short, deacylated tRNA samples were ligated to an annealed adapter set (first adapters) that is complementary to the 3′ NCCA tRNA overhang and contains short poly(A) tail, using T4 RNA ligase 2 (New England Biolabs) for 2 h at room temperature. Then, DNA oligonucleotides with identical sequence to ONT RTA adapters (second adapters) were annealed and ligated using T4 DNA ligase (New England Biolabs) for 30 min at room temperature. The ligated tRNA samples were converted into cDNA using Maxima H Minus Reverse Transcriptase cDNA synthesis kit (Life Technologies). After each ligation step, annealed tRNAs were cleaned and recovered using CleanNGS kit (CNGS-0050, GC biotech) according to the manufacturer’s protocol, measured on nanodrop and their profiles were confirmed using Agilent 2100 Bioanalyzer, Small RNA analysis (Supplementary Fig. S1A).

Finally, the samples were ligated to either ONT RMX sequencing adapters according to SQK-RNA002 kit (ONT) protocol or to RNA Ligation Adapter (RLA) according to SQK-RNA004 kit (ONT) and recovered using CleanNGS kit (CNGS-0050, GC biotech). The library was prepared for loading according to protocol then loaded on the appropriate RNA MinION Flow Cell (RNA002 or RNA004).

### qRT-PCR

For qRT-PCR analysis of tRNA expression levels, tRNAs were extracted and deacylated. Then, as described in “library preparation for tRNA sequencing,” deacylated tRNA samples were ligated to first and second adapters, then converted into cDNA using Maxima H Minus Reverse Transcriptase cDNA synthesis kit (Life Technologies). qPCRs to determine tRNA expression levels were performed using the primers listed in Supplementary Data Table S1. The qPCRs were performed using the SensiFAST SYBR No-ROX kit (Bioline) on a Quantstudio 5 machine (Applied Biosystems). Data were analyzed by use of the ^ΔΔ^Ct method.

### Shotgun RNA mass spectrometry

To assess the modification status of OHyW derivatives at position 37 of tRNAPhe, class I tRNA was gel purified from small RNA fraction. Shotgun mass spectrometry of RNase A-digested tRNA fragments was performed as previously described [23] with minor modifications. Briefly, 125 ng of the purified class I tRNA was digested with 10 ng of RNase A (Invitrogen, AM2271) in 20 mM ammonium acetate (NH4OAc, pH 7.0) at 37°C for 1 h. The resulting fragments were subjected to capillary liquid chromatography (LC)/nano-electron spray ionization mass spectrometry (ESI-MS) using DiNa splitless nano HPLC system (Techno Alpha) coupled with an Orbitrap Eclipse Tribrid mass spectrometer (Thermo Fisher Scientific). The digests were loaded on a trap column (L-column2 ODS, 5 μm particle size, 0.3 × 5 mm; Chemical Evaluation and Research Institute) and subsequently separated on a HiQ sil C18W-3 nanospray column (C18, 3 μm particle size, 0.1 × 100 mm; Techno Alpha) using a linear gradient of 2.5%–50% methanol containing 0.4 M 1,1,1,3,3,3-hexafluoro-2-propanol (pH adjusted to 7.0 with triethylamine) over 35 min at a flow rate of 300 nl/min. Mass spectra were acquired in the *m/z* range of 600–2000. The relative abundance of each modified species was calculated based on the peak heights of extracted ion chromatograms corresponding to RNA fragments with or without the target modification, using Qual Browser module in Xcalibur 4.2.28.14 software (Thermo Fisher Scientific).

### MinION Sequencing and base-calling

Nanopore sequencing runs were done using Minknow version 23.11.7 for a period of 72 h using the default settings except for disabling “reserve pores.” Raw results were stored as POD5 and live-base-calling was disabled. Base-calling was performed with dorado version 0.8.0 (https://github.com/nanoporetech/dorado) using the HAC model version 5.1 combined with the modifications for m^5^C, m^6^A, and pseU and stored as unaligned BAM files. The modification probability tables are stored in the SAM tags for every read.

### Total tRNA and Isodecoder Reference Curation

Human tRNA sequences derived from the hg19 reference genome along with the annotated conserved positions were extracted from the tRNAviz source data (http://trna.ucsc.edu/tRNAviz/ [24]). This human tRNA collection contains 414 sequences distributes across the genome and can be reduced to 267 unique tRNA sequences coding for the 47 different isoacceptors. The reference sequence for the alignment was made by padding the unique tRNA sequences with the adapter sequences used in library construction as this improved the number reads that could be aligned by the aligner.

### Alignment of human tRNA reads

After base-calling alignment was performed to the unique set of tRNA reference sequences using BWA-MEM aligner with the following arguments -h30,200 -W13 -k6 -xont2d -T20. Because Burrows–Wheeler Aligner (BWA) does not accept input sequences from unaligned BAM, and the modification data are present in the BAM tags, a custom wrapper was used to feed and collect the read data to BWA (https://github.com/NKI-GCF/trnamaprecal).

### Alignment mapping quality adjustment

The reference sequences are relatively short and contain tRNA sequences that differ as little as 1 nt from other sequences. Because BWA considers multi-mapping reads when the alignment scores are within 80% of the primary alignment, many reads are reported with a mapping quality of 0. Many of these reads do show a higher score for the primary alignment and the difference is often as high as the distance in the reference sequence would allow. To still be able to use these reads, the mapping qualities are modified by comparing the difference in alignment score from the primary to the best alternative alignments. If the difference in alignment score is >0 the mapping quality is set to this value if it is higher than the originally reported mapping quality (mapq) (Supplementary Fig. S1B). This functionality is implemented together with the codon quantification in a custom program (https://github.com/NKI-GCF/trnamaprecal).

### Analyses of tRNA expression levels

Anticodon expression level counts were quantified from the read data. Only reads mapping to the forward strand with a quality score >4.0 are used. For quality control purposes counting is performed per adjusted mapq value. When the mapq is 0 the read is quantified as “same target” or “same codon” when multiple alignments exists with the same alignment score. Counting is performed simultaneously with the mapping quality adjustment and the results are stored in text files. For creating the expression matrix the values of mapq > 0 and the “same codon” mappings are summed.

### Adjustment of MinKNOW configuration for SQK-RNA002 kit (ONT) runs

For comparison with previous described method [16], MinKNOW parameters were adjusted in a similar manner. The sequencing was ran without live base-calling and the bulk dump raw file was recorded. MinKNOW version 21.06.0 was used for sequencing and running the simulations, with either default and alternative MinKNOW configuration. With default configuration, adapter duration is defined as up to 5 s and the sequenced read (strand) as at least 2 s. The alternative configuration shorten adapter duration to 1 s and the strand duration to 2 s that results in higher number of aligned and uniquely aligned reads (mostly short reads gained). Subsequently, the number of reported, basecalled, aligned and uniquely aligned reads generated by default and custom MinKNOW configurations were compared.

### Modification annotation

Positions for conserved modifications for *Homo sapiens* tRNAs were manually curated using published literature and MODOMICS database (https://iimcb.genesilico.pl/modomics/) [25–27].

### Base-calling error rate analysis

Evidence for tRNA modifications was derived from base-calling errors. Base pileup counts were generated using Rsamtools (https://doi.org/10.18129/B9.bioc.Rsamtools), using reads with a minimum (adjusted) mapping quality of 1, a minimum basecall quality of 2 and coverage range of 50 000. The reference base fraction was calculated by dividing the count for the reference base by the sum of the counts for the canonical bases and the deletions. The relative error for any position was calculated as the number of the incorrect basecalls divided by the total count of the sequencing events (match/mismatch/deletion). Only position with at least 80 counts were included. Experimental versus control error ratios were calculated by taking the log_2_ of the ratio between the average base-error for the experimental condition replicates and the average base-error of the control replicates. The global error change analysis was performed by running multiple *t*-tests for all conserved (Sprinzl) positions by grouping together the relative error rate of all anticodon sequences for each condition. The resulting *P*-values were adjusted for multiple testing using Benjamini–Hochberg procedure.

### m^5^C, pseudouridine (Ψ), and m^6^A modification analysis using Nanopore’s Modkit tool

Modkit version 0.4.1 (https://github.com/nanoporetech/modkit) was used to filter and pile up the called modifications of the Dorado basecaller. Reads were filtered to a minimum mapping quality of 1 and a filter-percentile of 0.4 was used. The pileup files were analyzed using R. Reported modified sites were filtered for calls on the plus-strand and only calls that were present when the reference base matches the unmodified basecall were kept. Modified sites were included only if they have a minimum of 100 valid calls (confidently called as modified or unmodified). Modification probabilities for each site were estimated by averaging the base modification frequencies of all untreated samples and replicates. The sites were subsequently filtered for modification probabilities of > 0%, >10%, or > 20% and used for comparison with both UBS-seq and BACS. UBS-seq positions were extracted from the paper’s supplemental data [28] and re-annotated according to Sprinzl tRNA annotation. For UBS-seq m^5^C sites, all reported modified sites were included. For Ψ sites identified by BACS, only modified positions exceeding 10% were included (defined in [27] as “high-confidence Ψ sites”).

## Results

### Improved human tRNA detection using RNA004 chemistry

The most recent development of nanopore technology is the RNA004 chemistry. This chemistry includes a unique feature that enables high-depth sequencing of shorter RNA reads (∼50 nucleotides). In comparison, the former chemistry, RNA002, was limited in its ability to detect RNA molecules shorter than 100 nucleotides. Thus, we set out to examine whether implementing RNA004 chemistry to nanopore tRNA-seq will improve sequencing yield. For comparison with previously established methods [16, 17], library preparation was performed similarly, either with RNA002 or RNA004, with adjustments according to the used kit. In brief, RNA was extracted from melanoma cancer cell lines (A375 and SK-MEL-28) and the tRNA fraction was isolated. Then, the first pre-annealed adapter set that complements the mature tRNA 3’CCA overhang was ligated to the tRNA fraction, followed by the second adapter set ligation (Fig. 1A), and the ligation efficiency was confirmed using Bioanalyzer (Supplementary Fig. S1A and Supplementary Data Table S1). To finalize library prep, tRNAs were ligated to either RNA002 or RNA004 sequencing adapters (RMX or RLA in accordance) and loaded on the appropriate RNA MinION Flow Cell (Fig. 1A).

After base-calling, reads were aligned using BWA-MEM algorithm to the unique set of tRNA reference sequences. Alignment of the obtained human tRNA reads is challenging due to their relatively short length, abundant modification sites, and limited diversity in the reference sequences [17]. These issues need careful consideration when employing BWA, a short-read mapping algorithm. BWA produces a mapping quality (MAPQ) which, in a genomic alignment, predicts the probability that the alignment was placed incorrectly. To determine mapping quality, the algorithm uses the difference in alignment score between primary and alternative alignments as a base, and then applies several corrections, including penalizing for the number of alternative mappings. BWA reports multi-mapping reads when the alignment scores are within 80% of the primary alignment. Consequently, the significant similarity between tRNA genes results in many reads being assigned a mapping quality of 0 and cannot be mapped. To address this issue, we examined all alternative mappings and established a modified mapping quality (MOD-MAPQ), calculated as the difference in alignment scores between the primary alignment and the best alternative alignment (*AS*_primary_*– AS*_best_alt_, Supplementary Fig. S1B). If the difference in the alignment scores is >0 and exceeds the originally reported MAPQ, the mapping quality is set to this value. Reads with MAPQ ≥ 1 were assigned to tRNA anticodons, as well as reads with MAPQ = 0 when all alternative alignments are to the same tRNA anticodon (Supplementary Fig. S1B).

For RNA002 sequencing data, the bulk raw dump file was recorded, then re-ran with an alternative configuration of MinKNOW- “simulation (SIM) run,” as described by [16] (“Materials and methods” section). These distinct parameters of the alternative configuration were reported to increase sequencing yield by capturing shorter tRNA molecules when compared to the nanopore default configuration. Indeed, a simulation run resulted in an average 5-fold increase in base-called reads (raw output) (Supplementary Data Table S2), but only a 2.6-fold increase in mapped reads (Fig. 1B). Thus, while the number of base-called reads increased, the proportion of proper (sense) mapping was reduced by almost half, from 59.5%–69.5% to 29.2%–39.4% (Fig. 1C and Supplementary Data Table S2).

Next, we compared the yield of RNA002 runs with the improved alternative configuration (RNA002-SIM) with data acquired using the new RNA004 chemistry with the default configuration. Interestingly, the new RNA004 sequencing approach yielded, on average, a 3-fold increase in base-called reads (Supplementary Data Table S2) and a 6.4-fold increase in mapped reads (Fig. 1B). Therefore, the fraction of properly mapped reads was increased >2-fold from 34.5% on average to 74.6%. Conversely, antisense fraction was reduced by 6.6-fold (Fig. 1C and Supplementary Data Table S2).

Finally, the reproducibility of the data between replicates was assessed using both sequencing methods of RNA002-SIM and RNA004. While both methods yielded highly replicable data, RNA004 sequencing showed a higher correlation between replicates in both tested cell lines. For the A375 cell line, the correlation coefficient was elevated from 0.858 to 0.982 (Fig. 1D and E; Supplementary Fig. S2A andSupplementary Data Table S3), and for SK-MEL-28 from 0.896 to 0.996 (Fig. 1F and G; Supplementary Fig. S2A andSupplementary Data Table S3).

Previous work by Lucas *et al.* [16] indicated that the reverse transcription (RT) step enhances sequencing yield when employing RNA002 with an alternative configuration. This improvement was attributed to the linearization of tRNA, which increases translocation rate and improves sequencing accuracy. To evaluate the necessity of the RT step in RNA004 tRNA-seq, we used libraries prepared both with and without the inclusion of the RT step. The results indicated no significant difference in the proportion of correctly mapped reads, antisense mapped reads and unmapped reads between “with RT” and “without RT” samples (Supplementary Fig. S2B and Supplementary Data Table S4). The relative abundance of tRNA isoacceptors also remained largely comparable during the RT step, exhibiting a high correlation across samples in both replicates (*R* = 0.96 and 0.99; Supplementary Fig. S2C and Supplementary Data Table S4). Thus, while the overall results obtained with and without RT appear to be relatively similar, additional work is necessary to determine whether RT step can be excluded when conducting tRNA-seq with RNA004.

Altogether, our results indicate that tRNA-seq using RNA004 surpasses RNA002 in both sequencing yield and quality, and data reproducibility. Furthermore, RNA004 significantly shortens the sequencing and data acquisition workflow as there is no longer a need to alter the default settings, record, and re-run bulk raw data files.

### Identifying expression differences in tRNA anticodon pools using RNA004 tRNA-seq

**Figure 2.**
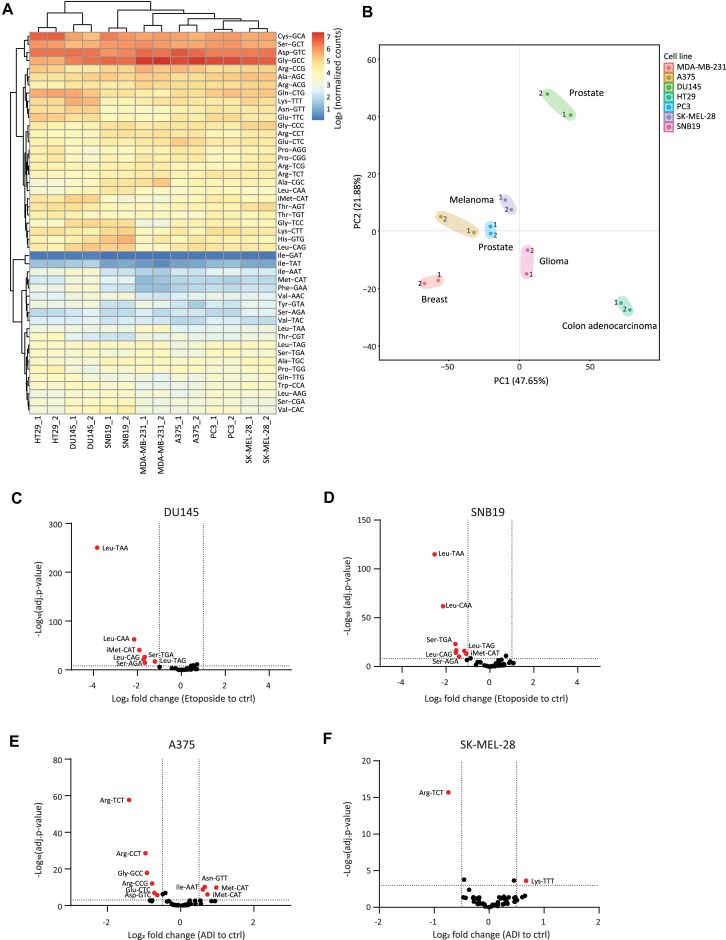
(**A**) Heat map of log_2_ tRNA anticodons expression across cell lines. Two biological replicates of each cell line shown. (**B**) PCA plots of tRNA anticodons expression for each cell line used. Two biological replicates for each cell line are shown and cell types are mentioned. (**C** and **D**) Volcano plots depicting differentially expressed tRNA anticodons in etoposide treatment condition relative to control using DESeq2. Marked in red are significant tRNAs with adjusted *P*-value below 10^−8^ and absolute log_2_ fold change above 1. (**E** and **F**) Volcano plots depicting differentially expressed tRNA anticodons in ADI treatment condition relative to control using DESeq2. Marked in red are significant tRNAs with adjusted *P*-value below 0.001 and absolute log_2_ fold change above 0.5.

While, generally, tRNA anticodon pools were found to be stable across human tissues [13, 29], alterations in tRNA expression were observed across cancer types, mediated via activation of oncogenic pathways such as PI3K/AKT/mTOR, and MYC, which regulate RNA polymerase III-mediated tRNA expression [8, 28]. These alterations were linked to variations in codon usage, presumably to fine-tune mRNA translation to match the altered demand of the cancer cells. We sought to test differences in the levels of tRNA isoacceptors expression in a panel of cancer cell lines from diverse cancer types using RNA004 tRNA-seq. Reads that could be uniquely assigned to a specific anticodon were counted and normalized to a fixed sum of 1000 counts. As shown in Fig. 2A, general similarities of tRNA expression were observed across cell lines, with tRNA^Ile^(GAT) as the lowest expressed tRNA, and tRNA^Gly^(GCC), tRNA^Asp^(GTC), tRNA^Ser^(GCT), and tRNA^Cys^(GCA) as the highly expressed tRNAs. Principal component analysis (PCA) and *Z*-score analysis, though, have identified variations in the tRNA expression repertoire between cell lines (Fig. 2B; Supplementary Fig. S3A and Supplementary Data Tables S5 and 6). Understanding the differences in tRNA expression and how they correlate with variability in cell types requires further investigation and validation.

Next, we evaluated the proficiency of our method in detecting variations in tRNA levels in response to stress stimulations. It was shown that Schlafen family member 11 (SLFN11), a type II tRNA endonuclease targeting tRNA^Leu^ and tRNA^Ser^ in general, but with high cleavage specificity toward tRNA^Leu^(TAA), is activated by DNA damage agents such as etoposide [30–33]. To test whether RNA004 can capture such changes, we treated the SLFN11-expressing DU145 prostate cancer and SNB19 glioblastoma cells with etoposide and subjected them to RNA004 tRNA-seq. The results confirmed significant downregulation of type II tRNAs following treatment, with tRNA^Leu^(TAA) showing the most significant reduction in both cell types. Downregulation of tRNA^iMet^(CAT), a type I tRNA that decodes ATG codon for translation initiation, was also detected following etoposide treatment (Fig. 2C and D; Supplementary Fig. S3B and Supplementary Data Table S7). This phenomenon, previously documented in other research findings, may be attributed to distinct characteristics of this tRNA relative to other type I tRNAs [30, 34].

To further inspect the sensitivity of our method, we tested cells treated with arginine deprivation, as another stress condition. Arginine is a semi-essential amino acid whose production in many cancer cells is inhibited by the suppression of Argininosuccinate Synthase 1 (ASS1), a key enzyme in arginine biosynthesis. While this endows cancer cells with a better proliferation capacity and resilience to stress [35–40], it also makes them auxotrophic to arginine. We therefore depleted ASS1-low melanoma cell lines (A375 and SK-MEL-28) from arginine via treatment with arginine deiminase (ADI), an enzyme catalyzing the degradation of arginine, and subjected total RNA to RNA004 tRNA-seq. As shown in Fig. 2E and F, we surprisingly observed that only the tRNA^Arg^(TCT) isoacceptor was significantly reduced in both cell lines (Supplementary Fig. S3C and Supplementary Data Table S8). To validate this result, we employed qRT-PCR and, indeed, consistent with RNA004 tRNA-seq, demonstrated a significant reduction in tRNA^Arg^(TCT) levels following ADI treatment, whereas the control tRNAs, tRNA^Leu^(CAG) and tRNA^Trp^(CCA) showed no significant alterations (Supplementary Fig. S3D). The cause for the specific reduction of tRNA^Arg^(TCT) remains to be explored further.

Altogether, our results demonstrate that RNA004 tRNA-seq allows detection of changes in tRNA availability in naïve versus treated conditions.

### Modification annotations of tRNAs

Recent developments uncovered novel strategies for the detection of modifications on the native RNA strand sequenced using nanopore technology [41]. These strategies are based on two different approaches for analyzing the data. One is based on the quantification of base-calling errors (mismatch/indels) induced by modifications at a specific position [16, 17], while the other is based on *de novo* modification calling from the raw electrical signal, a feature that is now already incorporated in the updated base-calling and modification models for RNA004 [42–45]. Thus, we evaluated the capacity of both strategies to detect human tRNA modifications in our RNA004 tRNA-seq data.

### Strategy I: Inferring modifications using the base-calling error rate

**Figure 3.**
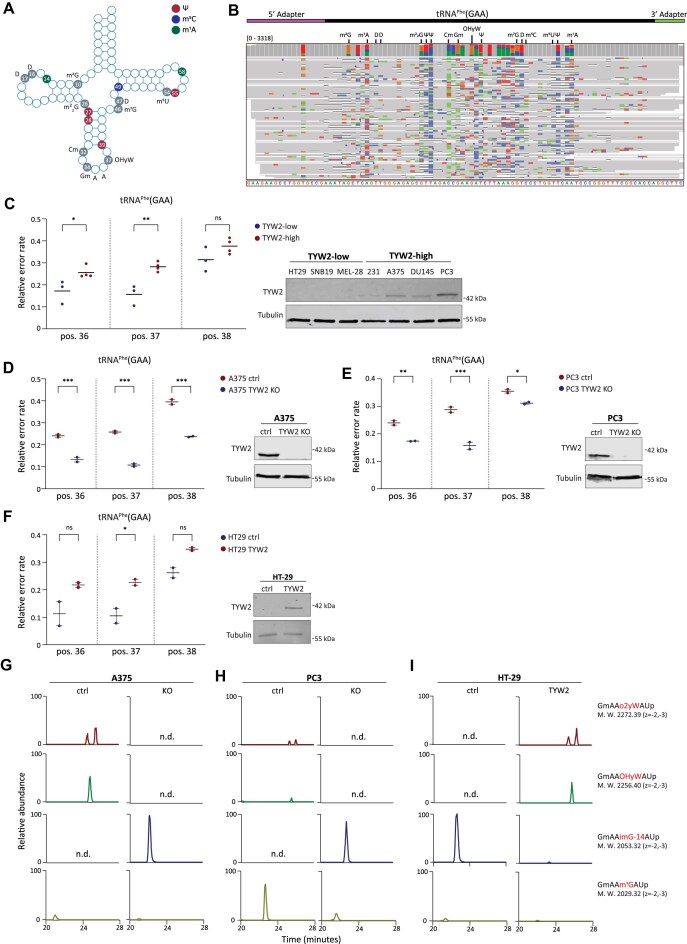
(**A**) Schematic representation of human tRNA^Phe^(GAA) sequence and its annotated modifications in literature. (**B**) IGV visualization of tRNA^Phe^(GAA) mapped reads aligned to the reference sequence. Positions colored are with a mismatch frequency higher than 0.2 and expected modification positions are indicated above. (**C**) (Right) western blot analysis of endogenous TYW2 levels in HT-29, SNB19, SK-MEL-28, MDA-MB-231, A375, DU145, and PC3 cells. Representative image shown from two biological replicates. (Left) bar graph showing relative error rate at positions 36-38 at tRNA^Phe^(GAA) in TYW2-high and TYW2-low cell lines. Data are mean ± s.d. of two biological replicates. *P < 0.033, **P < 0.002, ordinary one-way analysis of variance (ANOVA) using Sidak’s multiple testing correction. (**D** and **E**) (Right) Western blot analysis for TYW2 KO in A375 and PC3. Representative images shown from two biological replicates. (Left) The effect of TYW2 KO on relative error rate at positions 36–38 of tRNA^Phe^(GAA) in A375 and PC3. Data are mean ± s.d. of two biological replicates, *P < 0.033, **P < 0.002, ***P < 0.001 ordinary one-way ANOVA using Sidak’s multiple testing correction. (**F**) (Right) Western blot showing TYW2 levels and (left) bar graph presenting relative error rate of tRNA^Phe^(GAA) at positions 36–38 in HT-29 control cells and HT-29 with exogenous TYW2 expression. Data are mean ± s.d. of two biological replicates *P < 0.033, ordinary one-way ANOVA using Sidak’s multiple testing correction. (**G–I**) Extracted-ion chromatograms of RNase A-digested fragments of class I tRNAs containing position 37 of tRNA^Phe^ from A375 control and TYW2 KO cells (G), PC3 control and TYW2 KO cells (H) and HT-29 control and TYW2-expressing cells (I). Sequences of the detected fragments, with their molecular weights (M.W.) and charge states, are displayed on the right. Data presented are a representative replicate from three independent replicates.

Modifications of tRNAs can disrupt the signal of the expected current, potentially leading to base-calling errors (mismatch, insertion, and deletion). These base-calling errors can be traced back to known modifications, allowing the annotation of these modifications for the sequenced tRNA [16, 17]. To test this in our datasets, we examined tRNA^Phe^(GAA), one of the most studied tRNAs whose deregulation and improper modification are involved in many diseases, including cancer (Fig. 3A) [9, 10]. Using base-calling errors, we have successfully annotated the majority of the known tRNA^Phe^(GAA) modifications (13 out of 17) (Fig. 3B). Nevertheless, some modifications did not seem to induce robust miscalls, such as Dihydrouridine (D) in positions 16 and 17, ribothymidine (m^5^U) in position 54, and the conserved m^5^C in position 49, indicating that these modifications may require distinct approaches for detection. It is important to note that some sequencing errors, which cannot be readily explained, were also detected. These errors may arise from multiple factors, including base-calling errors resulting from neighboring modified sites, unknown modification sites, or genuine sequencing errors [19, 46, 47].

Amongst the annotated modifications is wybutosine modification (yW) (Fig. 3A and B), a large chemical modification found specifically in tRNA^Phe^(GAA) at position G37. yW modification promotes stabilization of codon–anticodon interactions, and its loss was found to provide tumor growth advantage and resistance to the cancer drug Taxol [10, 48]. While yW and its derivatives are biosynthesized by five enzymes, known as tRNA-yW Synthesizing Proteins 1-5 (TYW1-5) [49, 50], epigenetic silencing of TYW2 was found to be the leading cause for yW deficiency in cancer [10, 49]. Hence, we examined the link between TYW2 expression and modification-induced error rate at position 37, and its adjacent positions 36 and 38, as modification can potentially influence the current signal of its neighboring bases [16, 46]. The relative error for any position was defined as the number of incorrect base-calls divided by the total count of the sequencing events (match/mismatch/deletion). We examined TYW2-low cells (HT-29, SNB19 and SK-MEL-28) and TYW2-high cells (MDA-MB-231, A375, DU145, and PC3) and subjected them to RNA004 tRNA-seq. Subsequently, we analyzed the relative error rates at positions 36, 37, and 38 of tRNA^Phe^(GAA) (Fig. 3C and Supplementary Data Tables S9–15). Interestingly, the detected error rate correlated to a large extent with TYW2 expression, with a higher error rate detected in the TYW2-high cells, and most significantly in position 37, compared to TYW2-low cells (Fig. 3C).

To further validate the causal role of the TYW2 enzyme in modification-induced base-calling errors at these positions of tRNA^Phe^(GAA), we either knocked out TYW2 in the TYW2-high A375 and PC3 cells or ectopically expressed TYW2 in TYW2-low HT-29 cells (Fig. 3D–F). In line with our expectations, TYW2 knock-out (KO) led to a reduction in the error rate in these positions in both examined cell lines, A375 and PC3, with the most significant reduction in position 37 (Fig. 3D and E; Supplementary Data Tables 14 and 15). Accordingly, TYW2 expression in HT-29 induced a significant increase in error rate in position 37 (Fig. 3F and Supplementary Data Table S9). A global comparison using multiple *t*-tests was also applied to the relative error rate on all positions and anticodons between conditions. Although positions 36–38 were statistically significant only for A375 after correction for multiple testing (Supplementary Fig. S4A–C), these positions ranked highly across all comparisons (Supplementary data Tables S16–18).

To confirm the modification status of yW and its derivatives in tRNA^Phe^, we conducted liquid chromatography-mass spectrometry (LC-MS) shotgun analyses of tRNA modifications [23, 51]. The analyses confirmed that TYW2 KO, in both cell lines, led to loss of yW derivatives, hydroxywybutosine (OHyW) and peroxywybutosine (o2yW), and accumulation of TYW2 substrate, imG-14 (Fig. 3G and H; Supplementary Fig. S5A and B). In contrast, ectopic expression of TYW2 in HT-29 cells induced OHyW and o2yW accumulation and a reduction of imG-14 (Fig. 3I and Supplementary Fig. S6A).

In addition to yW, we also attempted to examine the effect of loss-of another tRNA-modifying enzyme, ubiquitin-related modifier-1 (URM1) [52]. URM1 functions as a sulfur carrier for tRNA thiolation on wobble uridines (U34) in the anticodons of tRNA^Arg^(TCT), tRNA^Glu^(TTC), and tRNA^Lys^(TTT) [53, 54]. However, no reduction in relative error rate was detected in these positions (Supplementary Fig. S6B and C).

A potential explanation for this failure is the small nature of the sulfur group (S_2_) added by URM1 on the previously added large modification, 5-methoxy-carbonyl-methyl on uridine 34 (mcm^5^U34) [55], thereby exerting too little effect on the nanopore current signal [46]. Altogether, our results suggest that inferring modifications using base-calling error rates is a promising approach for identifying cancer-associated tRNA modifications and their dynamics. However, it is less effective for detecting modifications that do not lead to significant base-calling alterations (false-negative).

### Strategy II: prediction of common tRNA modifications using signal analysis

As mentioned above, modified bases produce a different signal than unmodified bases. Interestingly, the new RNA004 chemistry is complemented with an updated Dorado base-caller, which offers a direct modification calling option from the raw signal [44, 45, 47]. Modification probabilities are recorded in the raw data during the sequencing run, and the pileup tool of the “modkit” software package, provided by ONT, is used to filter, call, and pile-up the modification data from Dorado. The resulting tables report the statistics of the different modifications at a base-pair resolution. The most important statistic is the “fraction modified” (modification probability), which is defined as the number of bases confidently called as modified divided by the total of the confident calls (modified and unmodified). For now, this feature is available only for common RNA modification, such as m^5^C, m^6^A, and Ψ. Thus, we used this feature to assess its ability to identify these modifications within sequenced tRNA molecules.

**Figure 4.**
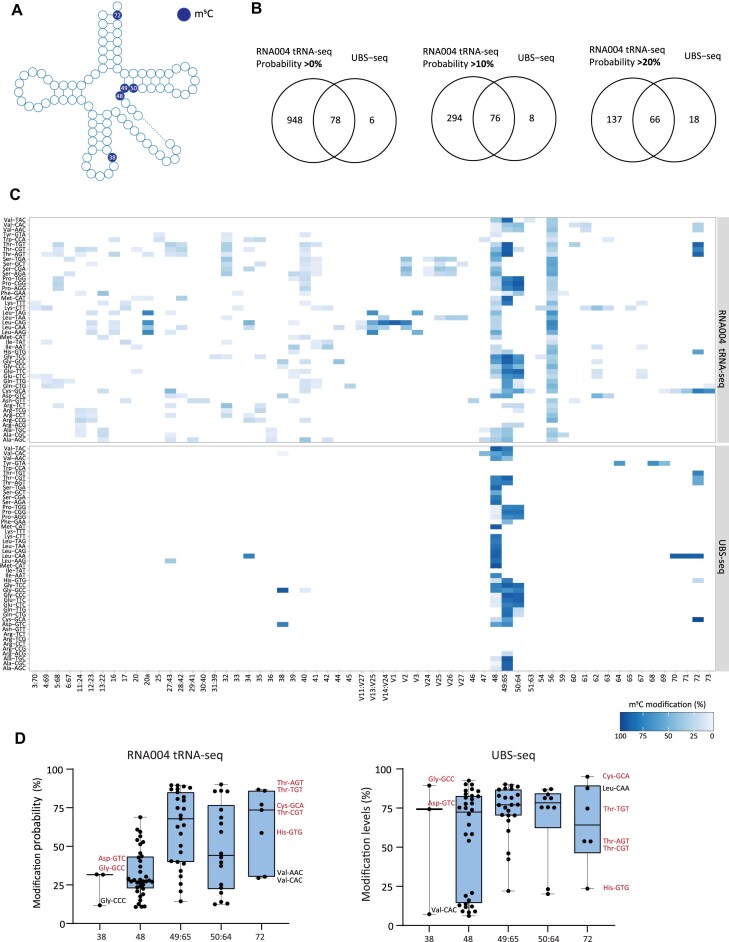
(**A**) Schematic of tRNA cloverleaf with annotation of conserved positions of m^5^C modification. (**B**) Venn diagrams illustrating the overlap of m^5^C sites identified by RNA004 tRNA-seq with varying probability thresholds (>0%, >10%, and >20%) and UBS-seq [28]. (**C**) Heat maps showing average modification rate (%) of m^5^C sites identified by RNA004 tRNA-seq (threshold of >10%, upper panel) and UBS-seq (lower panel). (**D**) Box plot showing average tRNA modification rate (%) at literature-annotated m^5^C sites [26] reported by RNA004 tRNA-seq with a minimum threshold of 10% (left) or UBS-seq (right). Each dot represents one tRNA isoacceptor. For positions 38 and 72, tRNA isoacceptors common to both methods are highlighted in red, while those specific to a single method are highlighted in black.

In Fig. 4A, we summarized the known positions of m^5^C modification in human cytosolic tRNAs, as indicated in the literature (Fig. 4A) [5]. To evaluate RNA004 tRNA-seq against an established method, we compared our results with the latest NGS technique, “Ultrafast Bisulfite sequencing” (UBS-seq), which maps m^5^C sites based on accelerated bisulfite reaction and C-to-U conversion [28].

**Figure 5.**
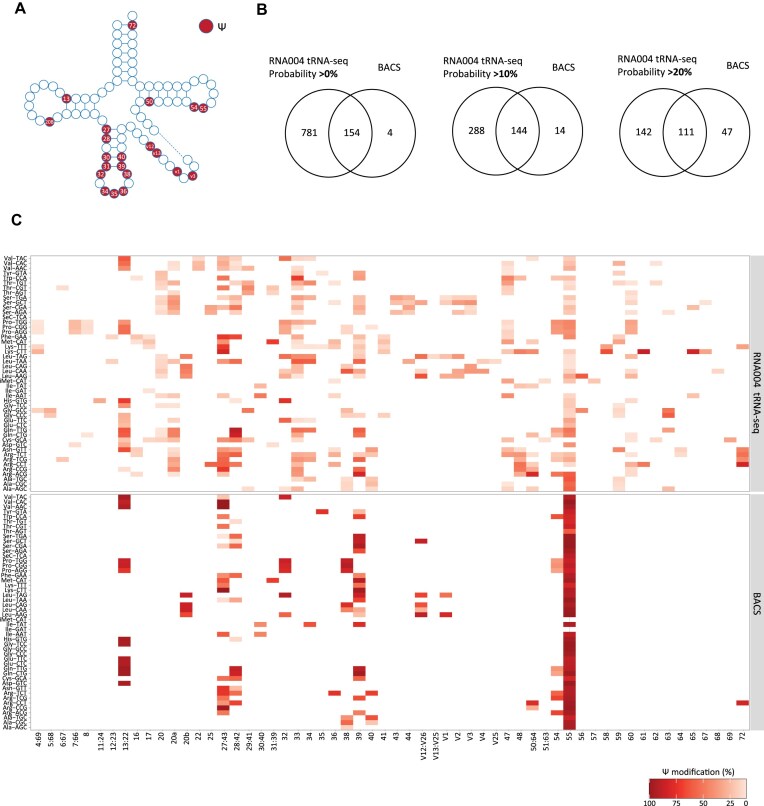
(**A**) Integrated view of Ψ modification profiles. (**B**) Venn diagrams illustrating the overlap of Ψ sites identified by RNA004 tRNA-seq with different probability thresholds (>0%, >10%, and >20%) and BACS [27]. (**C**) Heat maps showing average modification rate (%) of Ψ sites identified by RNA004 tRNA-seq with a minimum threshold of 10% (upper panel) and BACS (lower panel).

We considered RNA004 tRNA-seq sites with at least 100 valid calls and calculated the average modification probability across all wild-type cell lines. Subsequently, we compared positions with modification probability above 0% or 10% with UBS-seq-identified sites (Fig. 4B). While both cutoffs resulted in a comparable and substantial proportion of UBS-seq sites identified by RNA004 (∼90%, 78 or 76 sites out of 84, accordingly), cutoff of 10% seemed to significantly reduce potential background noise of nanopore-specific sites, from 948 to 294 (Fig. 4B). We then raised the cutoff to 20%, which further reduced nanopore-specific sites to 137. However, this adjustment also led to a considerable drop in the proportion of UBS-seq-identified sites, capturing only ∼78% of them (66 out of 84) (Fig. 4B). We therefore kept the 10% cutoff in further analysis to prevent the exclusion of potentially legitimate sites.

Next, we conducted a comprehensive comparison between the results of RNA004 tRNA-seq and UBS-seq. We focused on individual m^5^C tRNA sites and used literature annotation to select tRNA positions 38, 48–50, and 72 (Fig. 4A). Using both methods, m^5^C sites were predominantly identified across tRNAs at their highly conserved positions 48–50 (Fig. 4C and Supplementary Data Table S19). Methylation of these positions has been extensively studied and found to be catalyzed by NSUN2 (NOP2/Sun RNA methyltransferase 2) enzyme and to be important for tRNA stability [56–58].

Positions 38 and 72, on the other hand, show a less widespread modification pattern compared to positions 48–50, and appear specific to a subset of tRNAs, a finding supported by literature (Fig. 4C) [5, 59]. For position 38, both approaches identified a high modification rate in tRNA^Asp^(GTC) and tRNA^Gly^(GCC) (Fig. 4D and Supplementary Data Table S19). These tRNAs are the substrate of DNA methyltransferase 2 (Dnmt2) that catalyzes m^5^C formation at these positions [59]. In addition, both methods detected two other tRNA modifications at position 38, though to a lower extent. RNA004 tRNA-seq identified m^5^C at position 38 in tRNA^Gly^(CCC) with ~11% probability, while UBS-seq showed a 7% modification rate in position 38 of Val-CAC (Fig. 4D and Supplementary Data Table S19).

For position 72, both RNA004 tRNA-seq and UBS-seq reported high modification rate at specific tRNA types- tRNA^Thr^ and tRNA^Cys^ (Fig. 4D and Supplementary Data Table S19). This finding is supported by literature, as these tRNAs are known to be methylated at this position by the methyltransferase, NSUN6 [60]. Notably, both methods also detected m^5^C at position 72 of tRNA^His^(GTG), which might imply the potential discovery of a novel m^5^C site that, to the best of our knowledge, has not been previously annotated. It is important to note that variations between the methods at this position have also been observed. UBS-seq identified a high modification rate in tRNA^Leu^(CAA), whereas RNA004 tRNA-seq reported high modification probabilities in tRNA^Val^(AAC) and tRNA^Val^(CAC) (Fig. 4D and Supplementary Data Table S19).

As mentioned before, though nanopore recalled 90% of UBS-seq positions, it also detected another 294 sites (>10% cutoff) that were unidentified by UBS-seq. A notable difference is in the conserved C56, located at the TΨC-loop [61]. While RNA004 tRNA-seq detected high modification probabilities in this position across almost all tRNAs, UBS-seq did not identify it as a modified site (Fig. 4C and Supplementary Data Table S19). We consider this signal from RNA004 a false positive, potentially caused by signal interference from adjacent conserved Ψ sites. Alternatively, it may suggest the existence of a distinct conserved cytosine modification that nanopore is unable to differentiate from m^5^C. Another interesting divergence is observed at position 32, which seemed to be modified in serine, arginine, and threonine tRNAs (Fig. 4C and Supplementary Data Table S19). These tRNAs are known to have a N3-methylcytidine-32 (m^3^C) modification at this site [62, 63], a m^5^C isomer. This could suggest that the nanopore base-caller is likely unable to differentiate between the electrical signals of these isomers.

Similarly, analyzing m^6^A modification, we identified high modification probabilities at position 58 across tRNAs (Supplementary Fig. S7 and  Supplementary Data Table S20). However, this site is typically associated with a well-conserved m^1^A modification [5, 64]. m^1^A and m^6^A are chemical isomers that were previously reported to be indistinguishable in their electric signal, implying that RNA004 base-caller is limited in this aspect too [42].

We further examined the detection of Ψ, one of the most common modifications within human tRNAs, identified in >21 tRNA sites (Fig. 5A). For orthogonal validation, we conducted a comparison between our findings and those obtained from 2-bromoacrylamide-assisted cyclization sequencing (BACS), a recently developed chemical technique for quantitative sequencing of Ψ [27].

tRNA positions with at least 100 valid calls were considered and modification probability cut-off was set to 0%, 10%, or 20% (Fig. 5B). Similar to m^5^C, applying 10% cutoff on RNA004 data led to the identification of a significant proportion of BACS sites (∼91%, 144 site out of 158), while maintaining relatively low nanopore-specific sites (Fig. 5B). Thus, the threshold was set to 10% and tRNA modified sites, in both methods, were compared (Fig. 5C and Supplementary Data Table S21). The results indicated that conserved Ψ sites were dominantly identified in both methods at known positions 13, 27, 28, 54, and 55 (Fig. 5C and Supplementary Data Table S21). Interestingly, both techniques identified some tRNA-specific Ψ sites, including position 72 of tRNA^Arg^(TCT) and position 38 of tRNA^Leu^(CAA), increasing our confidence that these sites are truly modified (Fig. 5C and Supplementary Data Table S21). Of note, similar to m^5^C, discrepancies between the methods have also been observed, with 288 sites exclusively identified by RNA004 tRNA-seq. Hence, it is essential to further investigate the false-positive rate to improve nanopore modification-detection accuracy.

Thus, our results indicate that the new modification-calling feature of the updated Dorado base-caller has the potential to identify common tRNA modifications. However, it is still limited in several aspects, such as in distinguishing between chemical isomers, and its false positive rate should be thoroughly investigated for future applications.

## Discussion

In this study, we utilized new advances in nanopore RNA004 chemistry for the characterization of tRNA expression in human cells. Earlier methods employing the prior RNA002 chemistry could capture tRNAs, though generally with limited efficiency. Moreover, applying the recommended customized MinKNOW configuration [16] to improve read counts and to remove biases toward longer sequences increased sequencing yield but considerably reduced the proper mapping fraction. In contrast, implementation of the new RNA004 approach led to a 5-fold increase in the quantity of mapped reads, with up to 79% proper mapping. Additionally, the approach enhanced the reproducibility between samples. RNA004 tRNA-seq has also simplified sequencing procedures, as no setting adjustments, recording, and re-running bulk files were needed. The requirement for the RT step warrants additional investigation and evaluation.

Here, we tested the new RNA004 approach in detecting differential tRNA expression in a range of cell lines and conditions. Although limited in its extent, our data indicate differences in relative tRNA expression between cell lines, with each cell line expressing a unique tRNA repertoire. Whether the detected patterns of tRNA expression are causally linked to such cellular phenotypes remains to be investigated.

Previous studies established a connection between environmental stress conditions, such as chemotherapy and amino acid deprivation, and tRNA regulation [2, 32]. Indeed, our experiments using RNA004 tRNA-seq showed that exposure of cancer cells to the DNA-damaging agent, etoposide, led to downregulation of type II tRNAs and tRNA^iMet^(CAT), with the most dominant effect on tRNA^Leu^(TAA) as reported previously [30–33]. Moreover, subjecting cancer cells to arginine deprivation led to an intriguingly specific loss of tRNA^Arg^(TCT). This observation may hint at the involvement of a novel regulatory mechanism targeting specific tRNAs in stress conditions, such as tRNA-modifying enzymes that are important for tRNA stability.

tRNA modifications are key regulators of tRNA levels and activity in the cells, thereby facilitating optimal messenger RNA (mRNA) translation. Alterations in these tRNA modification profiles can lead to aberrant mRNA translation, which is linked to cell transformation and cancer progression [9, 65]. While the modification landscape of lower eukaryotic species, such as *Saccharomyces cerevisiae*, is almost completely resolved, the human landscape remains mostly uncharacterized [5]. Thus, to elucidate human cytosolic tRNA modifications in our RNA004 tRNA-seq data, we applied two nanopore modification-annotation strategies. The first strategy involves analyzing base-calling errors caused by modifications [16, 17], while the second incorporates a novel function of Dorado base-caller, complemented with RNA004, that reports site-specific probabilities of common modifications based on the raw signal [41, 46, 47].

We initially applied the base-calling errors strategy to examine the well-studied tRNA^Phe^(GAA). This analysis successfully annotated most of its modified positions. However, certain modifications, such as D at positions 16 and 17, m^5^U at position 54, and m^5^C at position 49, did not appear to cause significant miscalls. Among the annotated modification sites, we identified the cancer-associated yW modification at position 37. This was supported by a strong correlation between error rate levels at position 37 and its adjacent sites, with the expression of the main yW-modifying enzyme TYW2 [10, 66]. These findings were corroborated with LC-MS shotgun analyses of tRNA modifications. As the modification status of tRNA^Phe^ was linked to tumor growth advantage and chemotherapy resistance [7, 9, 10], our observations suggest that RNA004 tRNA-seq can potentially serve as a valuable tool for assessing the levels of cancer-related tRNA modifications.

Although the base-calling error rate strategy seems effective, it still suffers from several major drawbacks. First, it requires preceding knowledge of the tRNA modified positions; thus, in case of unknown modification sites, base-calling errors can be misinterpreted as false positives. Second, modifications might lead to base-calling errors at adjacent positions, as demonstrated with yW, making it difficult to accurately predict them at a single-nucleotide resolution. Third, not all modifications have a substantial effect on base calling errors, as evidenced by several tRNA^phe^ modifications and the failure to detect U34–tRNA thiolation [16, 17, 41, 46].

Some of these challenges might be addressed through the implementation of nanopore new modification-calling models for the Dorado base caller. Applying this new feature for m^5^C modification, we confirmed high m^5^C modification probabilities across tRNAs at the conserved positions 48–50 and at previously reported subsets of tRNAs at positions 38 and 72 [26, 28]. These results were corroborated with the orthogonal method for m^5^C detection- UBS-seq. Similarly, when examining Ψ, high modification probabilities were reported in its conserved positions and in a subset of tRNAs, which were also identified using BACS, an alternative Ψ-profiling approach [27].

Despite observed similarities with the orthogonal methods, RNA004 tRNA-seq reported a substantially higher number of modified sites, highlighting the challenges of efficient implementation of the new Dorado modification-calling feature [47, 67]. While differences might partly stem from variation in cell lines used between methods, high modification probabilities might suggest the presence of unidentified tRNA modification sites; however, these sites could arise from false-positive detections. The extent of false-positives is currently unknown and is particularly challenging due to the large diversity of RNA modifications, the lack of RNA-modification standards, and the incomplete modification-annotation of human tRNAs [47, 67]. Moreover, certain RNA modifications might not lead to changes in the signal that are sufficiently different from those caused by other RNA modifications [68]. Indeed, our findings suggest that the nanopore base-caller is limited in its capacity to differentiate between isomeric modifications. This was demonstrated at position 32, where we identified high m^5^C probabilities in a subset of tRNAs typically characterized to have m^3^C modification, an m^5^C isomer [62, 63]. A similar phenomenon was observed when applying the modification analysis of m^6^A. Research indicates that m^6^A tRNA-modified residues predominantly exist within archaeal and bacterial tRNAs [69]. Conversely, m^1^A is remarkably conserved within tRNAs of diverse species, including humans. m^1^A is typically found at position 58, mediated by the tRNA methyltransferases TRMT61A/TRMT6 complex and critical for tRNA stability and regulation [70]. Interestingly, our results indicated that the highest modification probabilities found across tRNAs were at position 58. This suggests that the nanopore modification calling algorithm, as other currently available algorithms [42], cannot differentiate the m^1^A and m^6^A isomers’ signals.

Altogether, further advances in the nanopore modification base-calling algorithm are needed in order to increase their accuracy and to expand the repertoire of possible detected modifications. Recent approaches aim to assess modification calling performance and establish thresholds for false positives [44, 45]; however, the prevalence of false-positive occurrences in tRNA molecules requires further examination. To ensure accuracy and reliability, validations of modification signals using knockouts or knockdowns of modifying enzymes, LC–MS/MS, and benchmarking with orthogonal methods will be required. Despite the challenges and limitations described above, our work demonstrated that the advanced RNA004 chemistry enables the detection of tRNA abundance and potentially modified tRNA residues in human cancer cells.

## Supplementary Material

zcaf044_Supplemental_Files

## Data Availability

Sequence data, tRNA expression counts, and modification pile-up data are available from GEO under accession number GSE291699. The software for the mapping quality recalibration and tRNA quantification is available on GitHub (https://github.com/NKI-GCF/trnamaprecal). The version used in this work is tagged as v0.1 and archived on Zenodo (https://doi.org/10.5281/zenodo.15011416). The reference sequence and the scripts for the analysis are also archived and available on Zenodo (https://doi.org/10.5281/zenodo.14989542).
