# Supplementary material for: Exploiting nanopore sequencing advances for tRNA sequencing of human cancer models
Source: NAR Cancer. 2025 Nov 3;7(4):zcaf044. doi: 10.1093/narcan/zcaf044 (PMC12582020; doi:10.1093/narcan/zcaf044)
Supplement: zcaf044_Supplemental_Files [file zcaf044_supplemental_files.zip › Supplementary_Figure_legends_Rev.docx]

**Supplementary Figure S1**

**a,** Bioanalyzer profiles of the tRNAs ligated to 1^st^ and 2^nd^ adapter sets and ran on nano RNA chip. **b**, Schematic overview of the tRNA analysis workflow and read alignment process.

**Supplementary Figure S2**

**a,** Correlation between tRNA abundances in A375 and SK-MEL-28 across replicates and RNA sequencing chemistries. Pearson’s correlation coefficient is indicated. **b,** Bar chart illustrating the proportion of properly mapped reads (blue), antisense reads (red) and unmapped reads (grey) relative to the total number of base-called reads obtained with RNA004 chemistry, with or without RT. Data are mean ±s.d. of two biological replicates. Ordinary one-way ANOVA using Sidak’s multiple testing correction. **c,** Scatter plots showing correlation of tRNA anticodons abundances of samples sequenced with RT step and without RT step. Data from two biological replicates are shown, and Pearson’s correlation coefficient is indicated.

**Supplementary Figure S3**
**a**, Heat-map shows the z-score of tRNA isoacceptors expression across examined cell lines. Columns are clustered based on Euclidean distance. **b-c**, Heat maps of Z scores and Log_2_ normalized counts of tRNA isoacceptors expression in SNB19 and DU145 cell lines in control and etoposide treatment conditions **(b)** and SK-MEL-28 and A375 cell lines in control and ADI treatment conditions **(c).** **d**, Bar plots representing the relative tRNA levels, acquired with qRT-PCR analysis, for control and ADI treatment in A375 cells. Data are mean ±s.d. of two biological replicates *** P <0.001, as per two-tailed t test.

**Supplementary Figure S4**
**a**, Heat maps of A375 (**a**) and PC3 (**b**) cell lines, displaying the log₂ fold change in error rate (TYW2 knockout versus control) with the corresponding Benjamini-Hochberg (BH) adjusted p‑values for each position analyzed across tRNA anticodons. **c**, Heat map showing the log₂ fold change in error rate of HT-29 TYW2-expressing cells versus control with the corresponding BH adjusted p‑values for each position analyzed across tRNA anticodons.

**Supplementary Figure S5**

**a-b,** XICs of RNase A-digested fragments of class I tRNAs containing position 37 of tRNA^Phe^ from A375 control and TYW2 KO cells (**a**), and PC3 control and TYW2 KO cells (**b**). Sequences of the detected fragments, with their molecular weights (M. W.) and charge states, are displayed on the right. Data from all three replicates are presented.

**Supplementary Figure S6**

**a,** XICs of RNase A-digested fragments of class I tRNAs bearing position 37 of tRNA^Phe^ from HT-29 control cells and TYW2-expressing cells. Sequences of the detected fragments, with their molecular weights (M. W.) and charge states, are displayed on the right. Data from all three replicates are presented. **b,** Western blot showing URM1 levels in A375 control cells and URM1 KO. **c**, Bar graph showing relative error rate of tRNA^Arg^(TCT), tRNA^Gly^(TTC), tRNA^Lys^(TTT) of wobble uridine at position 34. Data are mean ±s.d. of two biological replicates * P =0.04, as per two-tailed t test.

**Supplementary Figure S7**Heat map showing average modification probabilities (%) of m^6^A sites identified by RNA004 tRNA-seq with a minimum threshold of 10%. Highlighted in red is position 58, m^1^A conserved site.
