# Supplementary figures and images for: Exploiting nanopore sequencing advances for tRNA sequencing of human cancer models
Source: NAR Cancer. 2025 Nov 3;7(4):zcaf044. doi: 10.1093/narcan/zcaf044 (PMC12582020; doi:10.1093/narcan/zcaf044)

# Supplementary figure S1

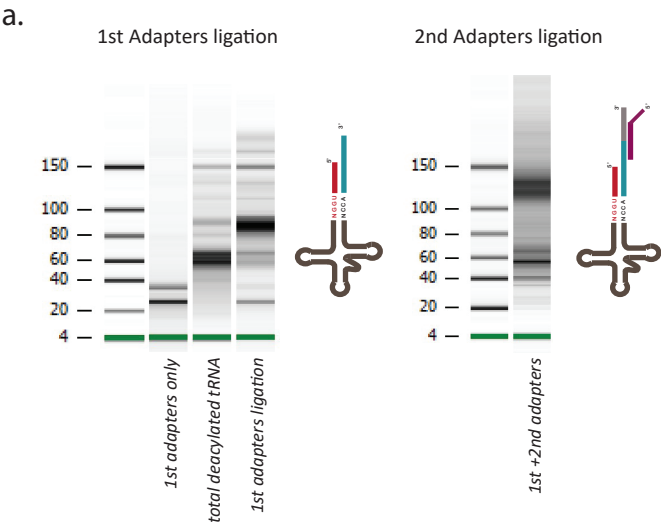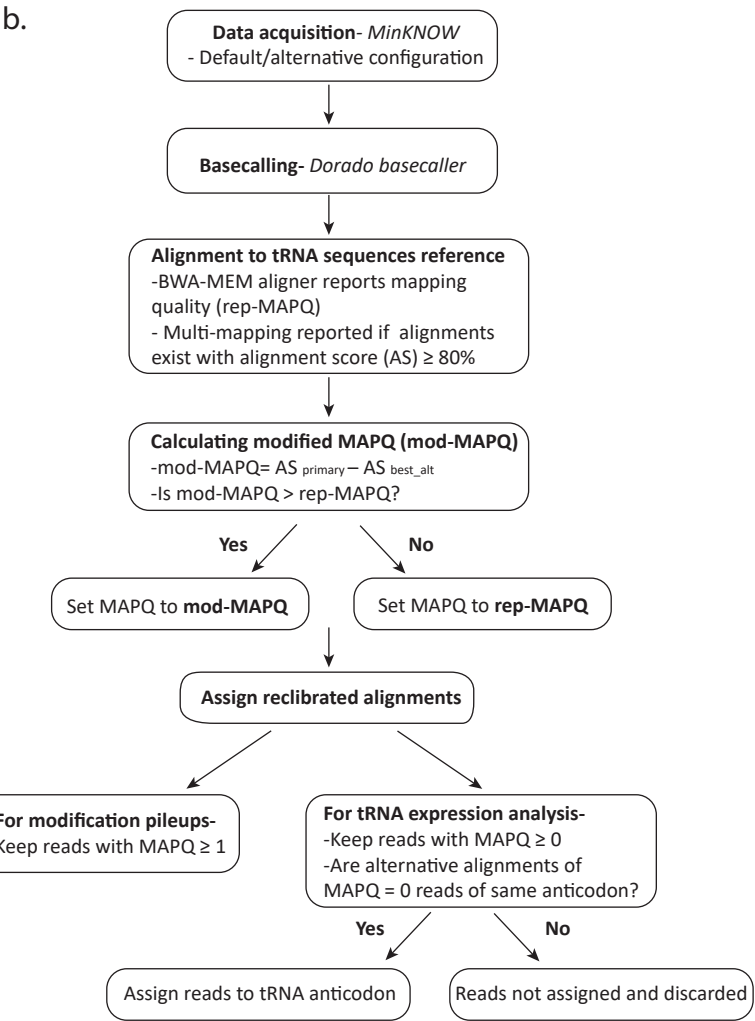

Supplement: zcaf044_Supplemental_Files [file zcaf044_supplemental_files.zip › Supplementary Figure S1.pdf]

Supplementary figure S2

a.

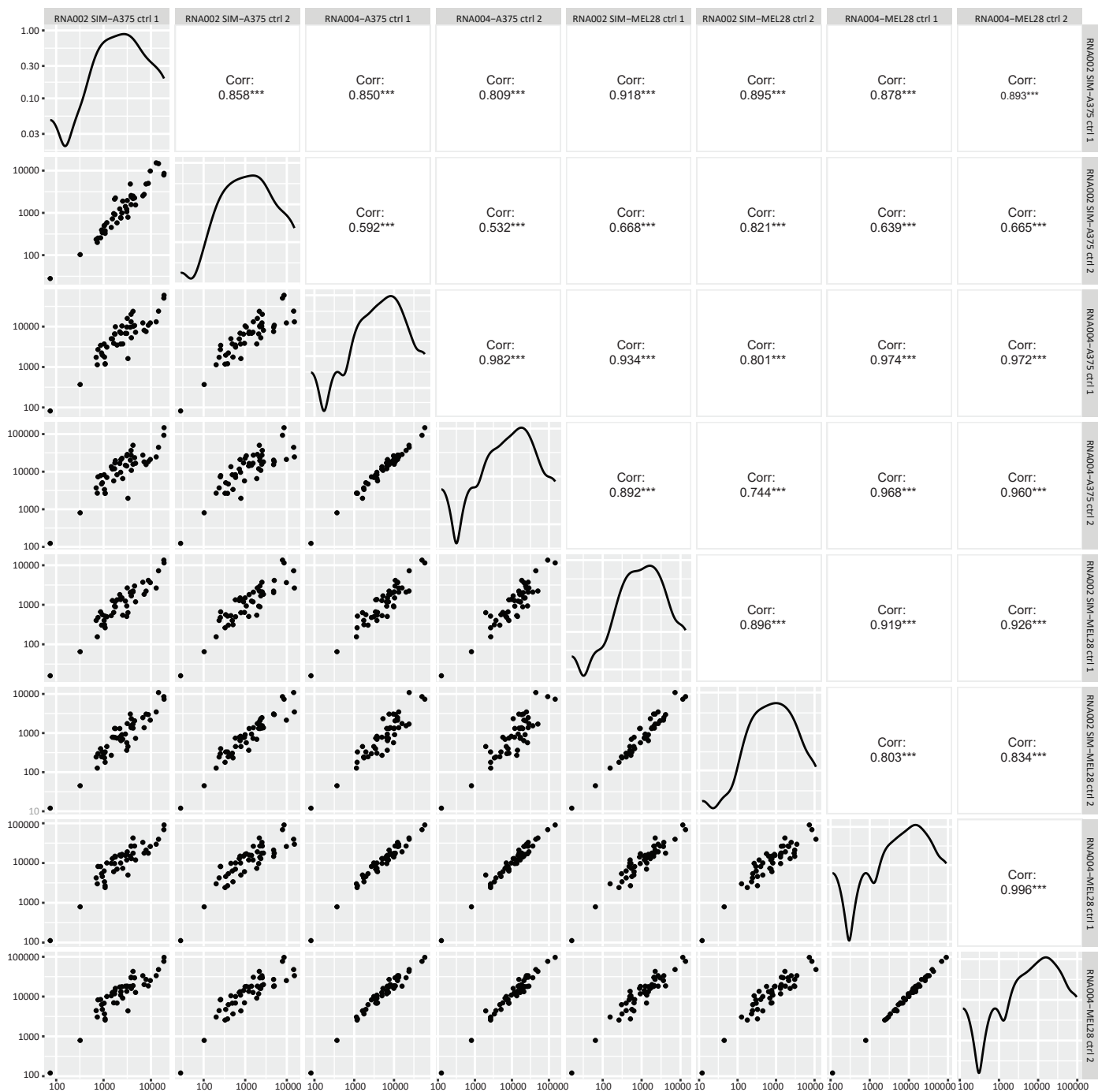

b.

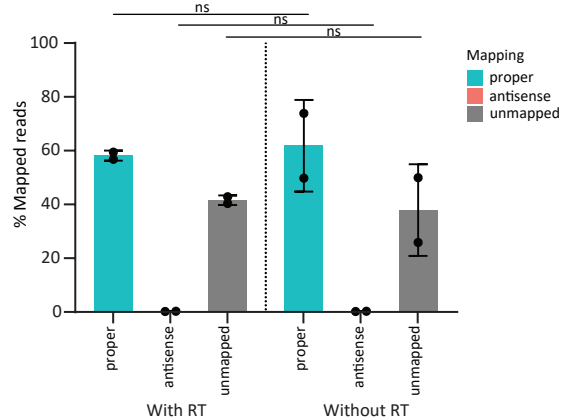

c.

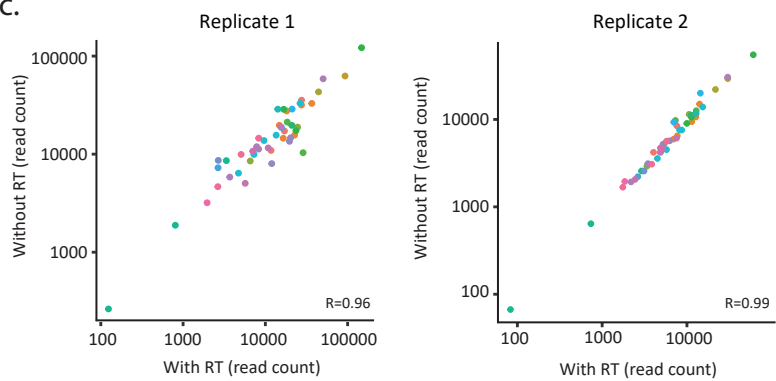

Supplement: zcaf044_Supplemental_Files [file zcaf044_supplemental_files.zip › Supplementary Figure S2.pdf]

Supplementary figure S3

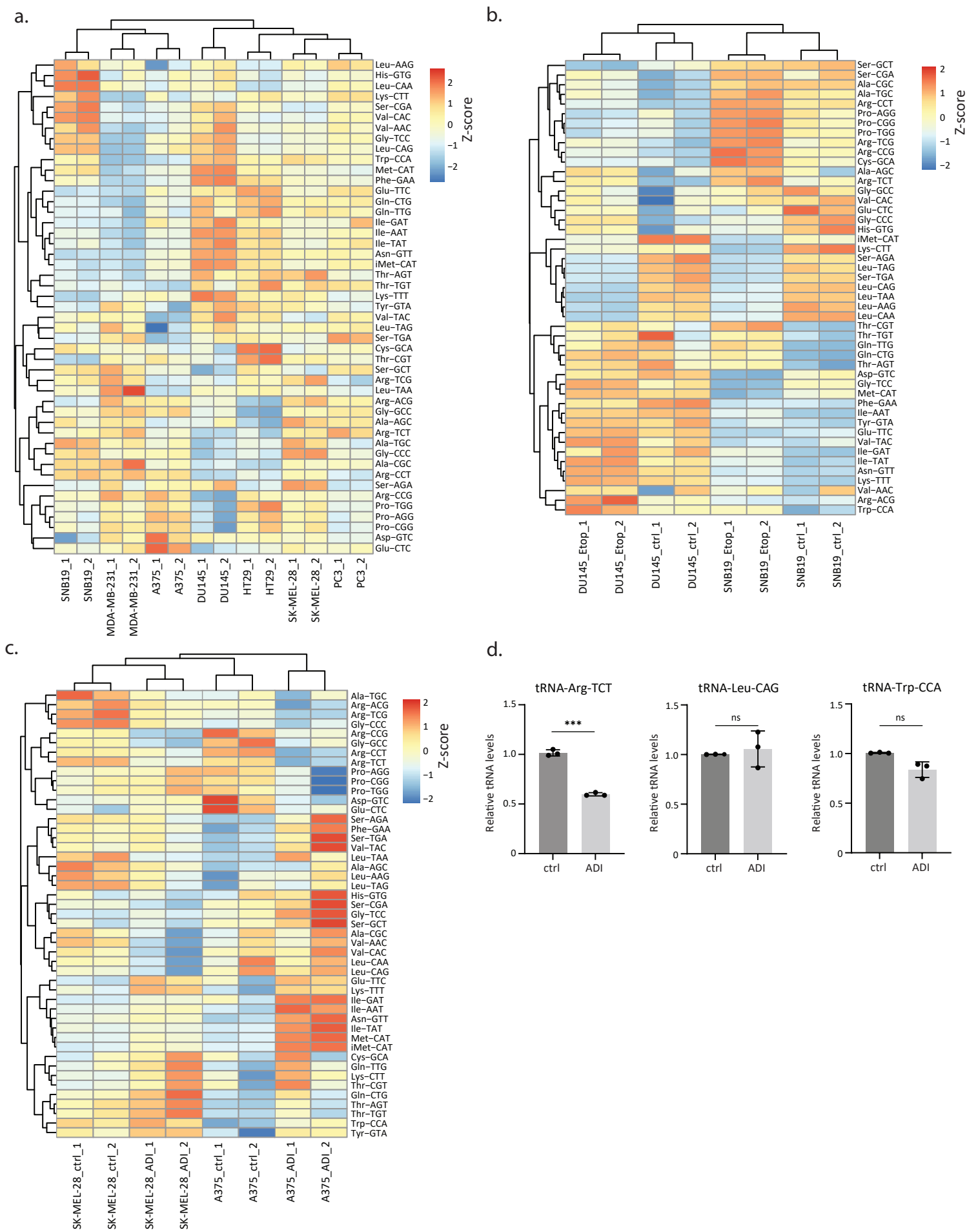

Supplement: zcaf044_Supplemental_Files [file zcaf044_supplemental_files.zip › Supplementary Figure S3.pdf]

Supplementary figure S4

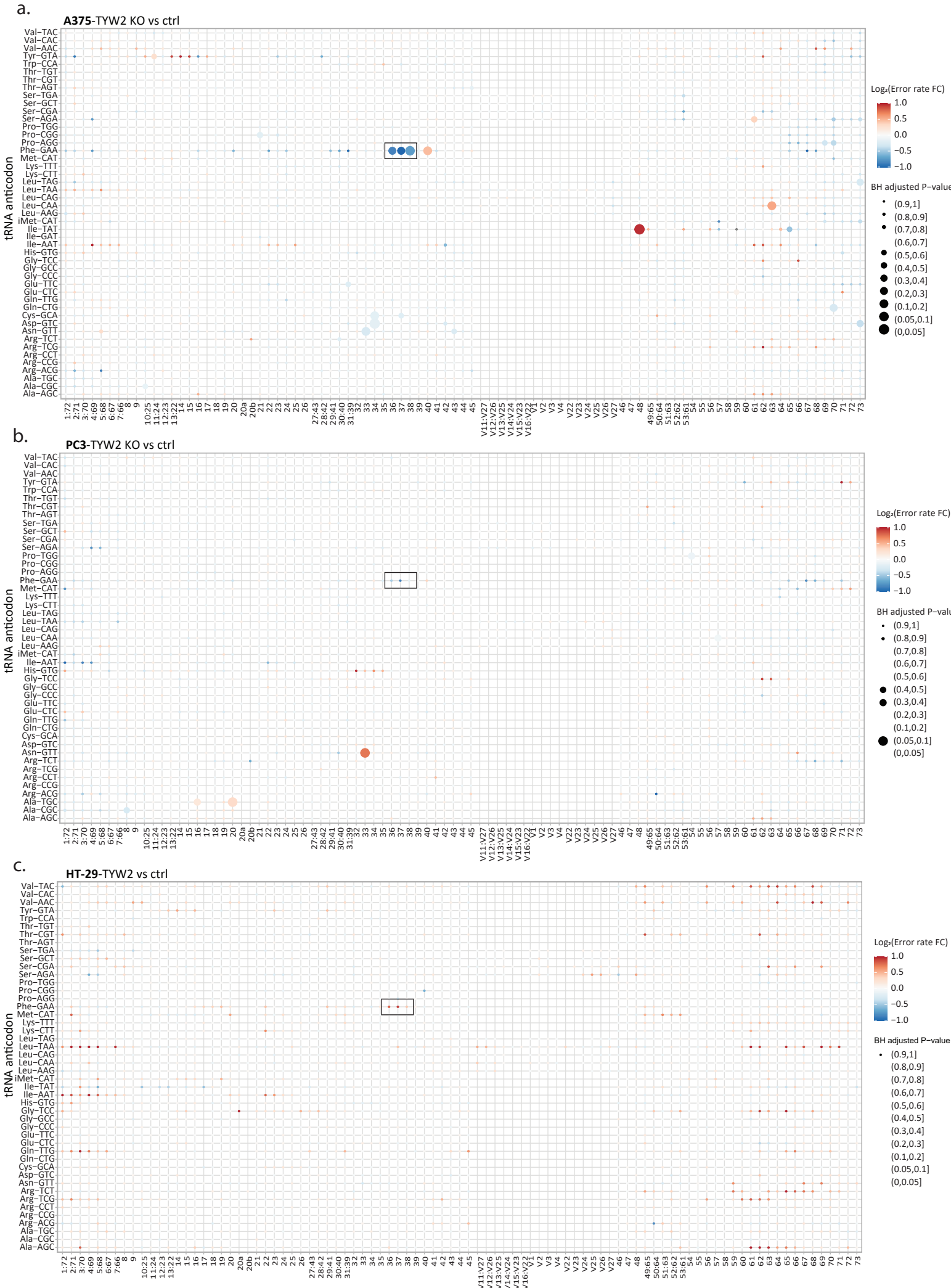

Supplement: zcaf044_Supplemental_Files [file zcaf044_supplemental_files.zip › Supplementary Figure S4.pdf]

Supplementary figure S5

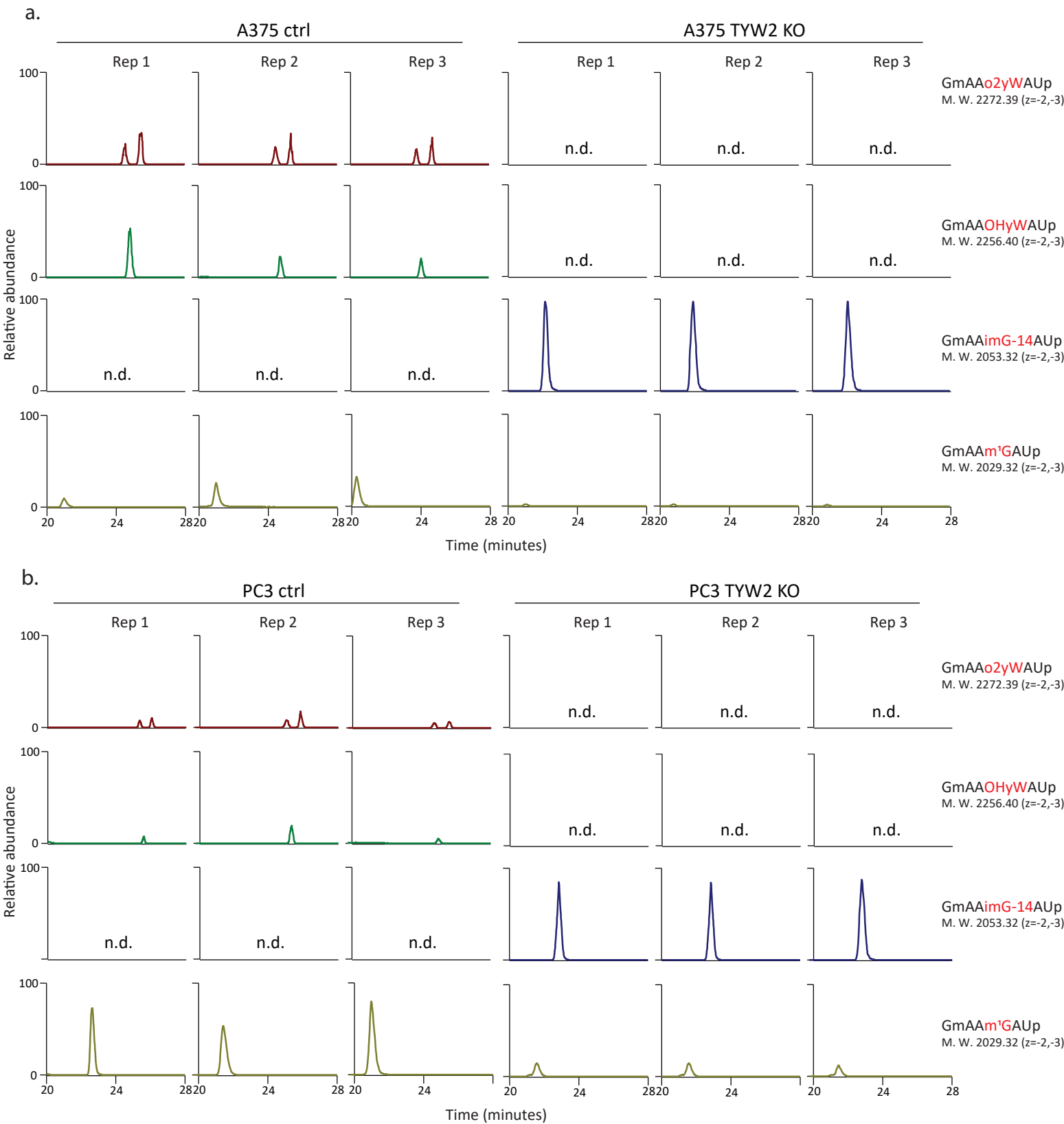

Supplement: zcaf044_Supplemental_Files [file zcaf044_supplemental_files.zip › Supplementary Figure S5.pdf]

Supplementary figure S6

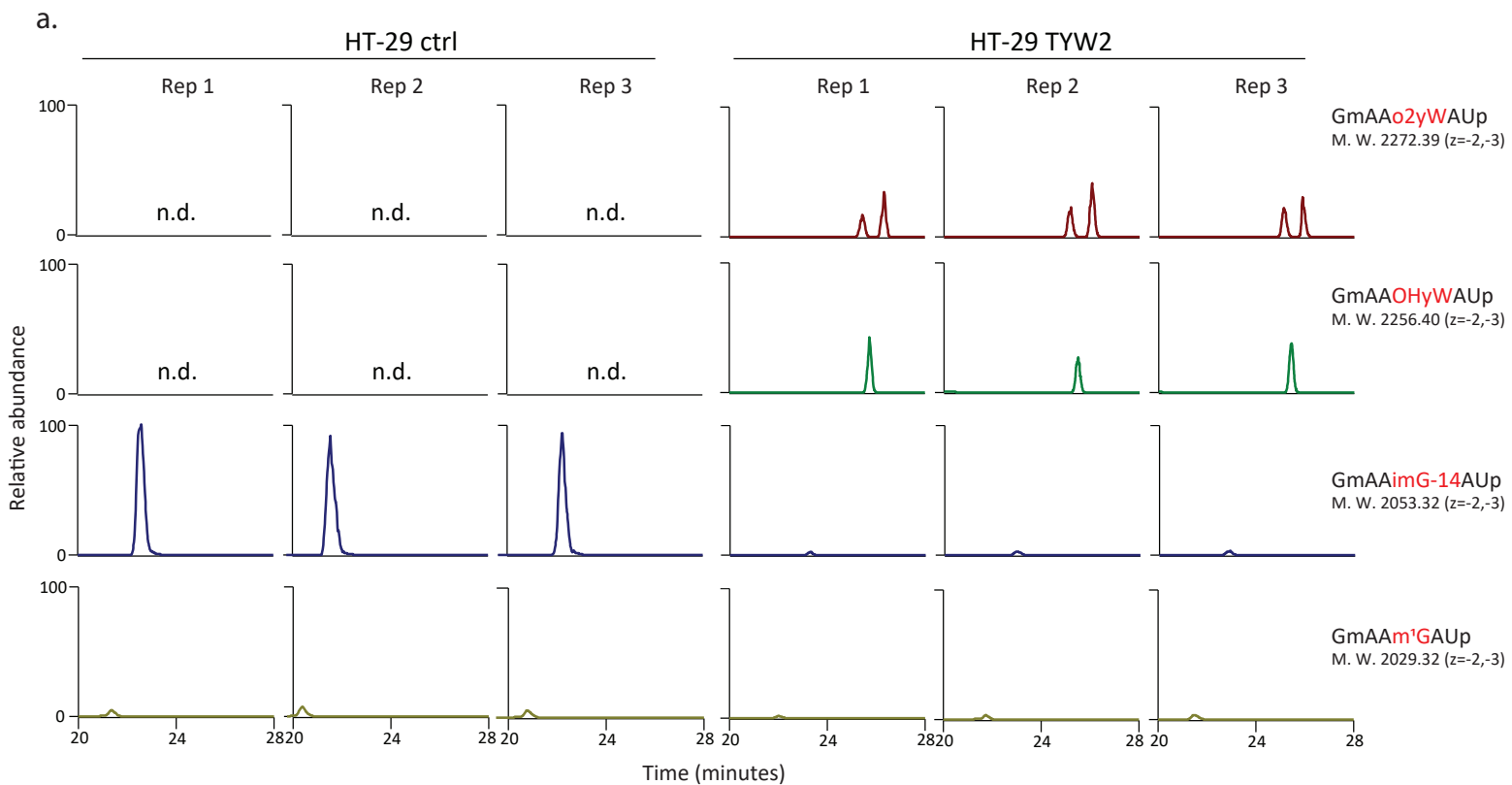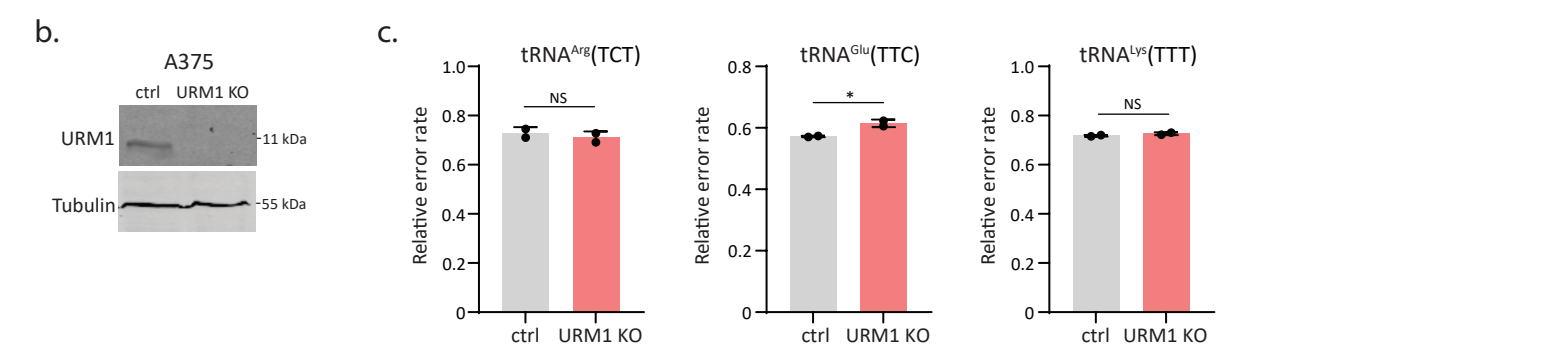

Supplement: zcaf044_Supplemental_Files [file zcaf044_supplemental_files.zip › Supplementary Figure S6.pdf]

## Supplementary figure S7

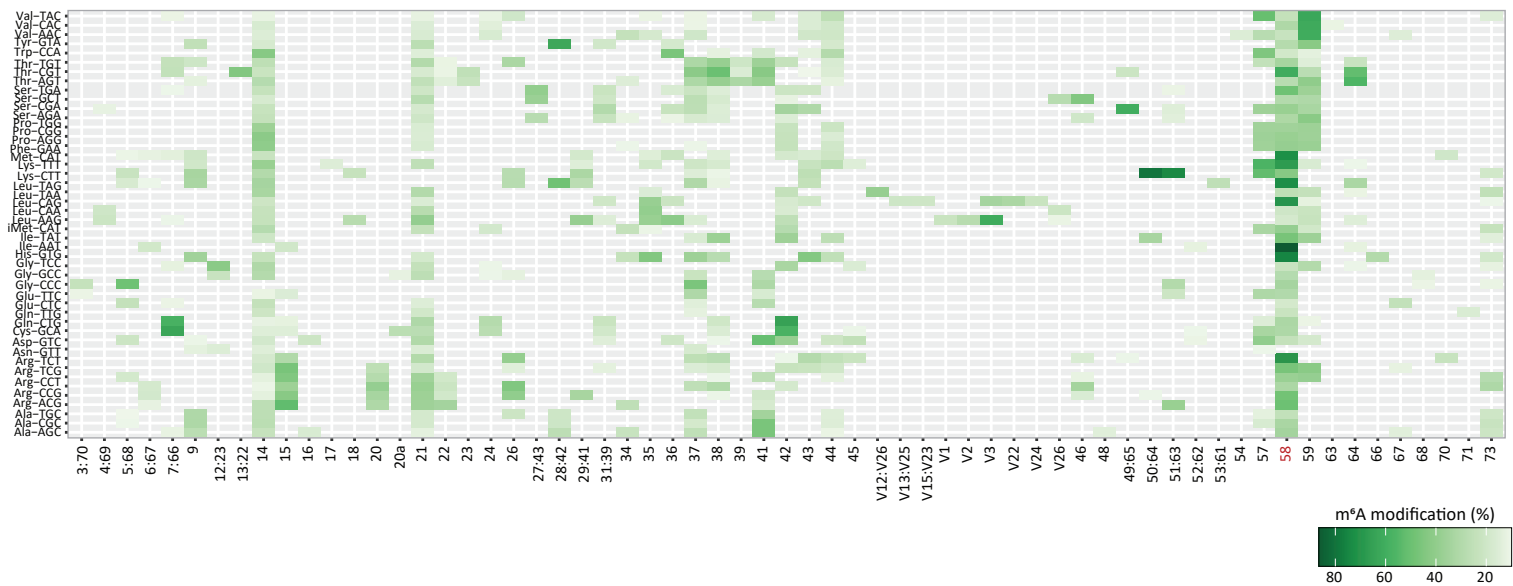

Supplement: zcaf044_Supplemental_Files [file zcaf044_supplemental_files.zip › Supplementary Figure S7.pdf]
